# Efficacy of a comprehensive strategy for the detection and treatment of hepatitis C infection in a population attending addiction centers

**DOI:** 10.3389/fpubh.2023.1092960

**Published:** 2023-02-02

**Authors:** Diana Corona-Mata, Antonio Rivero-Juárez, Ángela Camacho, Laura Ruiz-Torres, Inmaculada Ruiz-Cáceres, Bartolomé de la Fuente Darder, David Cáceres-Anillo, María de Guía Castro-Granados, María Lizaur-Barbudo, María Victoria Cabrera-Gisbert, Justa Redondo-Écija, Ana Aparicio-Aparicio, Leticia Manchado-López, Luciano Cobos, Ignacio Pérez-Valero, Antonio Rivero

**Affiliations:** ^1^Infectious Diseases Department, Maimonides Institute of Biomedical Research of Córdoba (IMIBIC), Reina Sofía University Hospital of Córdoba, University of Córdoba, Córdoba, Spain; ^2^CIBERINFEC, ISCIII – CIBER of Infectious Diseases, Instituto de Salud Carlos III, Madrid, Spain; ^3^Unidad de Drogas y Adicciones-CPD (UDA-CPD), Instituto Provincial Bienestar Social, Diputación Córdoba, Córdoba, Spain; ^4^Renacer Home, Córdoba, Spain

**Keywords:** hepatitis C, elimination, microelimination, drugs users, addiction centers, cirrhosis

## Abstract

**Background and aims:**

The burden hepatitis C infection in people with history or current drug use suppose a high risk of hepatic complications and transmission infectious disease. This population is poor linked to heath system and is difficult to achieve them and support treatment because they have high rates of lost follow-up. Our aim was to evaluate an intervention for the diagnosis and treatment of chronic hepatitis C and HIV in this population.

**Methods:**

Six-hundred and eighty-three people attended in Drugs and Addictions Centers (DAC) were asked to participate in health counseling and provide blood sample for test HCV, HIV, and syphilis from April 2019 to June 2020. Totally 556 subjects were surveyed and tested. All of them were assigned to a patient navigation program to improve health education and linking to the sanitary system. Hepatitis C infection patients were evaluated in an ampliated medical consult to evaluate hepatic stage with transient liver elastography and initiated Direct Acting Antivirals to achieve Sustained Viral Response.

**Results:**

Of the 556 patients who agreed to participate in the study, 33 (5.9%) had active HCV infection. Of the 33 patients infected with HCV, three were lost to follow-up once the diagnosis of HCV infection was made. Twenty-eight patients (93.3%) completed treatment and 26 achieved Sustained Viral Response (78.8%). Of the 30 patients, seven (23.3%) had advanced fibrosis, and of these, four (16.6%) had liver cirrhosis. One of the cirrhotic patients had hepatic space-occupying lesions at the baseline evaluation and was diagnosed with hepatocarcinoma.

**Conclusions:**

Our study suggests that the implementation of strategies based on personalized intervention models can contribute to the control of HCV infection in DAC users.

## Introduction

The high cure rates (>95%), the excellent tolerability profile of direct-acting antivirals (DAAs) and their universal use have drastically improved the prognosis of patients with chronic hepatitis C virus (HCV) infection (1). This scenario has allowed global programs to be launched with the objective of eliminating HCV by 2030 (2). One of the strategic objectives of these programs is to drastically reduce the rates of HCV infection in the most vulnerable populations, that is, those with greater risk of infection or disease progression (e.g., HIV infection, advanced liver disease, hemophiliacs, children, PWID). These strategies are known as microelimination strategies (3). Various actions have been carried out with the objective of achieving the microelimination of hepatitis C in some of these vulnerable populations, such as people living with HIV (PLWH) (4), individuals admitted to prisons (5) or people who are currently injected or have a history of injecting drugs (PWID) (6). Strategies aimed at individuals coinfected with HIV/HCV have been very effective because most of these patients are under active health control and monitoring (7). Strategies aimed at the microelimination of HCV in high-risk populations incarcerated in prisons have also shown high efficacy for reasons of accessibility and sanitary control during their admission to prison (8). However, microelimination strategies in PWID have been less effective, mainly due to the great difficulty of capturing and maintaining this population in the health system (9). The low efficacy of the strategies aimed at the microelimination of HCV in PWID has important consequences in the control of the infection. First, the difficulty in diagnosing and treating HCV infection increases the individual risk of liver disease progression (10). In addition, any untreated HCV-infected PWID is a reservoir of the virus that can cause the transmission and dissemination of the infection in its environment (11). Therefore, identifying new strategies to identify and treat HCV infection in PWID is a priority objective for the control of HCV infection.

Drug and addiction centers (DAC) treat patients with drug or toxic substance dependence and provide an excellent opportunity to access PWID patients. Different strategies focused on this opportunity have been designed and tested, including point-of-care strategies that include rapid antibody tests against HCV with subsequent referral to reference centers or test-and-treat strategies that include the dispensing of treatment in the centers (12–16). However, these strategies have not had the proposed efficacy and suffer from aspects as important as the comprehensive assessment of these patients (including the degree of liver fibrosis and the screening of advanced liver disease) and health education in risk prevention and diagnosis of comorbidities, among others. In this scenario, we designed a study to evaluate a strategy of supervised care of patients for screening, comprehensive evaluation (including screening for advanced liver disease) and treatment of HCV infection in users of DACs.

## Materials and methods

### Design and study population

This was a longitudinal prospective experimental study designed to evaluate an intervention for the diagnosis and treatment of chronic hepatitis C and HIV in a population of 12 DACs of the province of Córdoba (Andalusia, southern Spain) without direct access to HCV screening. These centers target all patients with drug or toxic substance dependence of Córdoba, city composed by 319.515, inhabitants. The study began in April 2019, and patient recruitment ended in July 2020. Patients treated in the DACs who met the following inclusion criteria were included: (i) over 18 years of age; (ii) in follow-up for one of their cessation programs. Patients under active follow-up by the Health System due to HIV infection or HCV infection were excluded.

### Intervention

The intervention strategy consisted of five steps.

#### Step 1: Patient navigator, education and multidisciplinary team

Candidate patients were identified in DACs by specialists in drugs and addictions. The information about them (including history of drug addiction) was transmitted by them to the Disease Service, where the study coordinator assigned a patient navigator responsible for the recruitment and monitoring of patients and provided risk assessment, health education, treatment adherence counseling, and medication coordination. To carry out this task, two patient navigators were hired (a nurse and a nursing assistant) with full dedication to the project. The patient navigators contacted each of the assigned patients to agree on a face-to-face appointment. This contact was repeated with at least three additional contact attempts for those patients who did not respond to the initial contact until the appointment was made. Patients who did not attend the scheduled appointment were repeatedly contacted until they attended.

A multidisciplinary and coordinated care plan was supervised by the study coordinator and designed for individuals involved in the strategy: addiction physicians, infectious disease specialists, hepatologists, social workers, pharmacists, nurses and patient navigators.

#### Step 2: Assessment and screening in a single act visit

The patients were treated in a specific consultation for a single act in which counseling for health promotion was performed, anthropometric data (weight, height) were obtained, blood pressure was measured, and blood analysis was performed for haemogram determination. Biochemistry, liver profile and serology of HCV, HIV and *Treponema pallidum*.

#### Step 3: Communication of results

All patients were informed by telephone of the results of the examinations performed. The information was also transmitted to the health personnel of the DACs. A new appointment was reconciled in a single medical consultation for patients diagnosed with HCV infection, according to the procedure expressed in point 1.

#### Step 4: Treatment of HCV, assessment of the degree of liver fibrosis and follow-up

Patients with HCV infection were clinically assessed in a single-act consultation that included the performance of transient liver elastography (TLE) and in which HCV treatment was initiated. The choice of treatment regimen was made at the discretion of the clinician (16–18). In patients diagnosed with hepatic cirrhosis, an analysis was additionally performed in the same act that included, among other determinations, levels of alpha-fetoprotein and albumin and a coagulation study. Hepatic ultrasound was planned, and patient follow-up was scheduled in coordination with the hepatology service of our hospital. In those patients who did not attend the scheduled appointment, the process of reconciliation of the appointment was repeated in collaboration with the DAC health personnel until they complied. All patients received treatment for 60 days following the initial visit.

After the start of treatment, a contact telephone number was provided to the patients for comments, questions or incidents, and a telephone follow-up was carried out at least every 2 weeks by the responsible tutor until the end of the treatment. In each telephone contact, adherence to treatment and possible adverse effects were evaluated and reinforced. A visit was planned at the end of treatment that included the determination of HCV-RNA. Additionally, patients were offered the possibility of making additional medical visits to reinforce adherence and identification of adverse effects; in these visits, HCV-RNA was determined.

#### Step 5: Assessment of sustained viral response

The sustained viral response assessment (SVR) visit was reconciled and scheduled at 12 weeks after the end of treatment. An active search was conducted for patients who did not attend the appointment for SVR assessment, and contact was repeated until their attendance was obtained.

### Variables and definition

The primary outcome variable of the study was SVR, defined by reaching undetectable HCV-RNA in serum 12 weeks after the end of treatment. The secondary variables were conducting HCV screening, defined as the completion of the single-act visit for patient assessment (Step 2), and initiation of HCV treatment, defined as taking at least one dose of the treatment prescribed by the clinician. End of treatment response (ETR) was defined as undetectable viral load after completion of therapy.

HCV infection screening was performed using a “one-step” diagnostic algorithm in which in all samples positive for antibodies against HCV, HCV RNA and its genotype were determined by quantitative techniques (19). Patients with a liver stiffness value >9.5 kPa were considered to have advanced fibrosis, and those patients with an RH value >12.9 kPa were considered cirrhotic (20).

### Statistical analysis

A descriptive analysis of the data was performed, expressing continuous variables as medians (Q1–Q3) and categorical variables as percentages (95% CI).

The primary outcome variable (SVR) was evaluated through the following analyses: (i) intention-to-treat analysis: All patients with HCV infection included in the study were included in the analysis, considering losses as failures of the strategy. (ii) Modified intention-to-treat analysis: All patients with HCV infection who received at least one dose of treatment were included in the analysis. (iii) Analysis by protocol: Only HCV-infected patients who completed the study protocol were included in the analysis.

The secondary outcome variable 1 was measured by the percentage of patients screened for HCV with respect to the population evaluated (number of people screened/total number of people evaluated × 100).

The secondary outcome variable 2 was measured by calculating the percentage of patients diagnosed with HCV infection who started treatment with respect to the total number of patients diagnosed (number of patients diagnosed who started treatment/total number of diagnosed × 100).

### Ethical aspects

This study was designed and performed according to the Helsinki Declaration. The Andalusian Ethical Committee approved the study protocol.

## Results

### Population

During the study period, 683 people met the inclusion criteria in the study, of whom 556 (81.4%) agreed to participate and 127 refused. The characteristics of the population identified according to whether they agreed to participate in the study are shown in Table 1. Three patients were identified with unknown HIV infection, one of whom was coinfected with hepatitis C. All patients presented asymptomatic HIV infection with lymphocyte counts <500 cells/ml and HIV-RNA >10,000 cop/ml. All patients started antiretroviral treatment. Nine patients with serological criteria for latent syphilis were identified and received treatment with penicillin G benzathine according to routine clinical practice.

**Table 1:** Descriptive analysis of the study population.

**Variable**	**Global**	**Participants accept screening**	**Participants do not accept screening**	***p*-Value**
*n*	683	556 (81.4%)	127 (18.6%)	
**Gender, (%)**				
Male	580 (84.9)	475 (85.4)	105 (82.7)	0.434
Woman	103 (15.1)	81 (14.6)	22 (17.3)	
Age (years). Median (IQR)	48 (39–56)	48 (40–57)	44 (37–52)	0.002
Smoker, *n* (%)	204 (29.8)	172 (30.9)	32 (25.2)	0.212
**Substance**, ***n*** **(%)**				
Alcohol	552 (80.9)	459 (82.7)	93 (73.6)	0.014
Cocaine	346 (50.7)	280 (50.5)	66 (52)	0.758
Cannabis	268 (39.3)	212 (38.2)	56 (44.1)	0.220
Synthetic drug	40 (5.9)	31 (5.6)	9 (7.1)	0.516
Opiates	154 (22.4)	118 (21.1)	36 (28.3)	0.077

*n*, number of individuals; IQR, interquartile range; *p*, *p*-value.

### Patients with HCV infection

Of the 556 patients who agreed to participate in the study, 33 (5.9%) had active HCV infection. The characteristics of these patients are shown in Table 2. Of the 33 patients infected with HCV, three were lost to follow-up once the diagnosis of HCV infection was made, before their clinical situation or the degree of liver fibrosis was evaluated and before HCV treatment. The characteristics of the 30 patients who continued in follow-up are shown in Table 2. Of the 30 patients, seven (23.3%) had advanced fibrosis, and of these, four (16.6%) had liver cirrhosis. One of the cirrhotic patients had hepatic space-occupying lesions at the baseline evaluation and was diagnosed with hepatocarcinoma.

**Table 2:** Characteristics of patients diagnosed with HCV infection.

**Characteristics**	**(*N* = 33)**
Age (years), median (IQR)	50 (46.5–57)
Male gender, *n* (%)	30 (90.9)
**Genotype**, ***n*** **(%)**	
1a	16 (48.5)
1b	1 (3)
3	4 (12.1)
4	5 (15.2)
Not genotyped	7 (21.2)
Degree of fibrosis, *n* (%)	30
Advanced fibrosis	4 (13.3)
Cirrhosis	5 (13.3)
Cirrhosis plus hepatocarcinoma	1 (3.3)
Treatment initiation, *n* (%)	30 (90.9)
Glecaprevir/pibrentasvir	15 (50)
Sofosbuvir/velpatasvir	15 (50)
Treatment outcome, *n* (%)	30 (100)
Loss to follow-up during therapy	2 (6.6)
Reach RFT	28 (93.3)
Loss to follow-up after ETR	2 (7.1)
Patients reaching SVR	26 (92.8)

Advanced fibrosis defined as Transient Liver Elastography (TLE) value 9–12.5 kPa; Cirrhosis defined as a TLE value > 12.5 kPa.

ETR, end of treatment response; SVR, sustained viral response.

### Rate of treatment uptake and SVR

Of the 33 patients diagnosed with active hepatitis C virus infection, 30 (90.90%, 95% CI 76.43–96.86%) started anti-HCV treatment. Of the 30 patients who started treatment, two were lost to follow-up, and 28 reached the end of treatment response (93.33%, 95% CI 78.68–98.15%). The two patients lost to follow-up were followed up and evaluated until week 4, presenting HCV-RNA of 17 and 826 IU/ml at this time. Of the 28 patients who reached ETR, 26 were evaluated at week 12 posttreatment, and all reached SVR. Therefore, the SVR rate obtained in the study was 78.78% (95% CI 62.25–89.32%) in the intention-to-treat analysis, 86.66% (95% CI 70.32–94.69%) in the modified intention-to-treat analysis and 100% (95% CI 87.13–100%) in the per-protocol analysis.

## Discussion

The screening strategy and HCV treatment support evaluated in our study achieved high rates of screening (81.4%) and SVR (86.6% in the modified intention-to-treat analysis) in DAC users. Our results suggest that a strategy based on a tutored intervention (patient navigator intervention), the work of a multidisciplinary team, patient education intervention and the use of the HCV diagnostic algorithm “in a single step” could be useful in the management of a population, such as DAC patients, in whom it is extremely difficult to make both the diagnosis and to achieve completion of the treatment.

Drug users who are unaware of being infected with HCV, in addition to being a serious health problem for individuals, is a serious public health problem and an important barrier to the control and elimination of HCV (21). The greatest difficulty in curing HCV infection among drug users lies in the difficulty of keeping them in treatment. Thus, Ford et al. (22) studied a high proportion (85%; *n* = 435) of patients with active hepatitis C (85%; *n* = 435) identified through a screening program. However, only six of them completed the treatment successfully. In this scenario, multiple strategies were identified to improve health care for people at high risk of hepatitis C infection (23). *Patient guardianship* has been associated with significantly increased adherence at the beginning and follow-up of treatment among patients with HCV infection compared to the standard of care in a randomized study, which included 1,353 patients (769 in the usual care group and 584 in the patient care group, respectively) (24). The guardianship group had significantly higher probabilities of adherence to care and initiation of treatment within the first 6 months. This study was the first to demonstrate that patient care compared to usual care increases the proportion of patients linked to health care. Our study supports the usefulness of this intervention in people with chronic HCV infection who use drugs.

The “one-step diagnosis of HCV” intervention used in our study is an intervention that simplifies and optimizes the diagnosis of HCV infection. RNA tests in the same sample used for the HCV antibody test significantly improved the acceptance of HCV RNA tests, reducing the number of visits for the patient and the time until diagnosis and minimizing the loss of patients during follow-up (25). For these reasons, this intervention should be considered an essential element in HCV screening strategies in populations with a high risk of infection and a low probability of adherence to the health system. Thus, this intervention has become the standard diagnostic method in Spain for hepatitis C (19). Patient education improves engagement in care in the context of other chronic diseases (26–29). Similarly, educational interventions regarding HCV infection and its treatment could increase the knowledge of the disease and, with it, the motivation of patients to be involved in their care. This could have contributed in our study to improving HCV screening rates and treatment adherence of infected patients. Another essential element of the strategy evaluated in our study was the coordination of the different health agents involved. This coordination is essential to optimize the effectiveness of health resources in the care of people who consume drugs because they reduce the fragmentation of care and facilitate the linkage of patients to them. The usefulness of this intervention has not been demonstrated in the context of HCV infection, but it has been observed in the context of other chronic diseases (30) and in drug users in a methadone maintenance program (31).

Whether the potential pharmacological interactions due to the consumption of drugs of abuse, alcohol or opioid replacement therapies could reduce the efficacy of HCV treatment with AAD is speculation (32). In our study, in the per-protocol analysis, 100% of patients achieved SVR. The four patients who started treatment and did not reach SVR were lost to follow-up (two of them after reaching ETR). These results support the findings of other studies that suggest that the response to HCV treatment with AAD in drug users can be comparable to that of the general population, provided that adequate adherence to treatment is ensured (33, 34).

On the other hand, it is important to note that 30% (*n* = 9) of patients with HCV infection in whom the degree of liver fibrosis could be assessed had advanced liver fibrosis at the time of diagnosis and that 16.6% had liver cirrhosis (one of them with hepatocarcinoma). The high percentage of patients with advanced liver disease observed in our study indicates the convenience of including a comprehensive assessment of patients, including the degree of liver fibrosis, in HCV screening and treatment programs in drug users. Some strategies, such as *test-and-treat*, suffer from this, despite the advantages of bringing screening closer to the comfort zone of the users. Therefore, strategies that do not include advanced liver disease screening are insufficient for the comprehensive management of hepatitis C in this population. This high percentage of patients with advanced disease, in turn, indicates the significant delay of the health system for the diagnosis of HCV infection in DAC users. Correcting this diagnostic delay is essential to achieve control of HCV both in this population and in the general population.

Our study has some limitations that require us to interpret its results with caution. First, this is a study conducted in DAC that did not have access to screening tests for HCV during the study. This meant that the initial phase of the strategy was focused on achieving the screening of a high proportion of patients. Therefore, our results cannot be extrapolated to DAC in other areas with access to HCV screening. However, in our health environment, access to HCV treatment is universal and free for the entire population, so our results cannot be extrapolated to areas in which access to HCV treatment for drug users is not guaranteed. Part of our study was conducted during the COVID-19 pandemic, and it is not possible to know the impact that this circumstance may have had on the results. Finally, our study was conducted in patients treated in DAC, and we have been able to access them by taking advantage of their link to these centers. Therefore, our results cannot be extrapolated to drug users who are not linked to the health system.

In conclusion, our study suggests that the implementation of strategies based on personalized intervention models can contribute to the control of HCV infection in DAC users.

## Data availability statement

The raw data supporting the conclusions of this article will be made available by the authors, without undue reservation.

## Ethics statement

The studies involving human participants were reviewed and approved by Ethics and Clinical Trials Committee (CEIC) of Andalusia. The patients/participants provided their written informed consent to participate in this study.

## Author contributions

AR and AR-J designed the study. BF, DC-A, MG, ML-B, MV, JR-É, AA-A, LM-L, and LC identified candidate patients in DACs and recruited individuals. DC-M, ÁC, IP-V, and AR were the patient reference tutors in the Infectious Disease Unit. DC-M, LR-T, and IR-C collected database. DC-M, AR-J, and AR analyzed statistics data, interpreted the results, and draft the paper. All authors revised the draft critically for important intellectual content, contributed to the article, and approved the submitted version.
